# The effect of visual scene on VEMP responses

**DOI:** 10.1007/s00405-026-10030-4

**Published:** 2026-02-16

**Authors:** Şeyma Tuğba Öztürk, Sema Satıcı, Cem Yeral, Zahra Polat, Oğuz Yılmaz

**Affiliations:** 1 Department of Audiology, College of Health and Science, Istanbul Medipol University, İstanbul, Turkey; 2 Audiology PhD Program, Institute of Health Sciences, Istanbul Medipol University, İstanbul, Turkey; 3 Department of Audiology, Hamidiye Faculty of Health Sciences, University of Health Sciences, Istanbul, Turkey; 4 Department of Audiology, Faculty of Health and Science, Istanbul Medipol University, İstanbul, Turkey; 5 Audiology PhD Program, Institute of Health Sciences, Istanbul University- Cerrahpaşa, İstanbul, Turkey; 6 Department of Otolaryngology, Umraniye Training and Research Hospital, University of Health Sciences, Istanbul, Turkey; 7 Department of Audiology, Faculty of Health and Science, İzmir Bakırçay University, İzmir, Turkey

**Keywords:** Vestibular evoked myogenic potentials, Visual perception, Postural balance, Otolithic membrane, Visual-vestibular interaction

## Abstract

**Purpose:**

To determine how visual motion direction and the engagement of central versus peripheral visual fields modulate cervical vestibular evoked myogenic potentials (cVEMP). We investigated whether optokinetic stimulation and smooth pursuit, delivered centrally or peripherally, alter cVEMP latency and amplitude.

**Methods:**

Forty-five healthy adults completed cVEMP testing across seven visual conditions: baseline (fixed gaze) and various optokinetic stimulation directions and smooth pursuit tasks. These stimuli were delivered using either a light bar (central vision, *n*=13) or an expanded visual surround (peripheral vision, *n*=32). Primary outcomes were P13 and N23 latencies and peak-to-peak amplitudes, with within-subject comparisons assessing deviations from baseline.

**Results:**

Central visual stimulation via the light bar produced no significant changes in cVEMP latencies or amplitudes relative to baseline (*p* > 0.008). In contrast, peripheral stimulation yielded significant latency prolongations, most prominently for N23 during vertical and horizontal optokinetic motion. P13 effects were less pronounced and inconsistent. Amplitudes did not show systematic changes across any condition. The divergent behaviour of P13 and N23 indicates differential susceptibility of early versus later cVEMP components to visual and cognitive influences.

**Conclusion:**

Peripheral visual motion exerts a more substantial influence on vestibulo-collic pathways than central foveal input, primarily by delaying cVEMP latencies without enhancing amplitudes. This pattern contrasts with prior vection-linked reports of latency reductions and amplitude increases, suggesting distinct neural processing for vection versus purely visual motion cues. These findings highlight the potential to leverage peripheral motion to modulate otolith reflex timing in vestibular rehabilitation, warranting confirmation in patient cohorts and with explicit vection quantification

## Introduction

The vestibular system plays a crucial role in maintaining balance, stabilising gaze, and ensuring spatial orientation [1]. To perform these tasks under varying environmental conditions, the balance system integrates sensory input from both external and internal sources. In this context, the balance system processes data received from somatosensory, visual, and vestibular structures in the inner ear. It produces immediate and rapid solutions to the interactions between these systems, and performs sensory reweighting when necessary [1]. However, visual stimuli often do not align with inputs from other sensory modalities. This incompatibility can cause disturbances, such as visually induced motion sickness [2], as well as the vection effect, which is also known as the motion aftereffect. Vection is the illusory sensation of self-motion triggered by visual stimuli and is influenced by factors such as stimulus realism and context [3–5]. In this regard, visual influences, particularly the effects of peripheral and central vision on the visual field, are known to impact the balance system. Berencsi et al. demonstrated that peripheral vision plays a more critical role than central vision in maintaining stable posture and that postural sway is more influenced by the direction of stimulus observation or head gaze than by the direction of trunk orientation. Accordingly, the study authors suggest that peripheral vision operates in a viewer-centred frame of reference, characterised by head/gaze direction, rather than a body-centred frame of reference, characterised by the anatomical planes of the body [6].

Among these balance structures, the vestibular apparatus, located in the inner ear, receives information about head movement via the angular semicircular canals and linear otolith organs of the head. Within these, the sacculus has a structure that detects linear accelerations in the vertical plane. The cVEMP test is currently used to evaluate the sacculus. This test begins with the transmission of saccule responses to an acoustic stimulus via the inferior vestibular nerve to the vestibular nucleus. At this main junction, all data (visual and somatosensory) are collected. Within the scope of these data, a motor response occurs, and sudden relaxations are observed in the sternocleidomastoid muscle located in the neck [5].

In this pathway, cVEMP responses may be affected by visual stimuli. In addition to Bonsu et al., our past studies have also shown that cVEMP amplitudes increase with visual motion in all directions [7, 8]. Dündar et al. also examined the relationship between vertical VR motion and cVEMP results, reporting an increase in amplitudes without a change in latencies [9]. While several studies report positive effects of visual stimuli on cVEMP amplitudes, Pöhlmann et al. argue that short-term interaction with video games can cause a temporary loss of cVEMP. That permanent loss of cVEMP can be detected in long-term video game players due to cybersickness [10].

In this case, it is not clear which types of visual stimuli have a positive or negative effect on cVEMP responses. The two possible factors here are the direction of movement (vertical, horizontal) and the question of how central and peripheral vision affect visual stimulation. This study aims to investigate how the direction of visual motion and the involvement of central versus peripheral vision influence cVEMP responses.

## Materials and methods

### Participants

A GPower analysis indicated a required sample size of 36 participants [44]. A total of 45 healthy adult participants, aged between 20 and 30 years, were recruited. Data from both ears (a total of 90 ears) were included. Participants reported no history of otological, ophthalmological, neurological, or psychiatric disorders. Experienced audiologists performed all procedures at … University Hospital and Centre for Audiology and Speech Disorders.

### Ethical considerations

Written informed consent was obtained from all participants prior to their inclusion [50]. The study protocol was approved by the Istanbul Medipol University Non-Interventional Clinical Research Ethics Committee (Decision No: 446, May 9, 2024), and all procedures were conducted in accordance with the Declaration of Helsinki.

### Experimental design

Participants were randomly assigned to either the central or peripheral visual condition using probability sampling to ensure unbiased allocation. No significant differences were found between the groups in terms of age and gender, ensuring comparability between conditions. A total of 13 participants (26 ears) were assigned to the light-bar condition (7 females, 6 males; mean age = 22.08 ± 1.83 years), while 32 participants (64 ears) completed the expanded visual field condition (18 females, 14 males; mean age = 22.19 ± 1.65 years).

The expanded visual field was assessed using a 43-inch (1,920 × 1,080 px) LED TV screen in the Micromedical VisualEyes 525 VNG device by Interacoustics (Interacoustics A/S, Middelfart, Denmark). In both conditions, c-VEMPs were recorded during a series of visually modulated tasks designed to manipulate visual motion input.

c-VEMP responses were acquired sequentially during the following seven visual conditions: (1) fixed gaze (baseline), where participants were instructed to fixate on a small target point at the centre of the blank LED screen in a dimly lit room; (2) leftward optokinetic stimulation; (3) rightward optokinetic stimulation; (4) downward optokinetic stimulation; (5) upward optokinetic stimulation; (6) smooth pursuit at 0.4 Hz; and (7) fixed gaze (retest). The stimuli were presented using both a light bar and an expanded visual field. The light bar was designed to primarily stimulate the central visual field, minimizing peripheral visual motion cues. In the expanded visual field condition, stimuli were delivered across a broader field of view to maximize peripheral visual input and enhance visual-vestibular interaction.

### VEMP recordings

All c-VEMP recordings were obtained using the Eclipse EP-25 system (Interacoustics A/S, Middelfart, Denmark). Each test was performed for both ears in a randomised order across participants. Electromyographic (EMG) activity was recorded from the ipsilateral sternocleidomastoid (SCM) muscle. For cervical vestibular evoked myogenic potentials, asymmetry was assessed following electromyography scaling. During all recordings, participants maintained tonic SCM contraction by [lifting their head against gravity/turning their head to the contralateral side]. The Eclipse system’s EMG monitoring feature was used to provide visual feedback, ensuring a consistent muscle contraction level (e.g., 50–100 µV) was maintained. Trials with insufficient SCM activation were rejected and repeated. c-VEMP recordings utilised a 500 Hz tone-burst stimulus at 100 dB SPL, delivered via insert earphones. For each condition, at least two recordings consisting of a minimum of 200 stimuli were acquired to ensure reliability. An asymmetry of 40 per cent or more was considered abnormal. The average of these recordings was used for further analysis.

### Data extraction and outcome measures

Recorded waveforms were visually inspected and analysed by experienced audiologists. For cVEMP, peak latencies of p13 and n23, along with p13-n23 amplitudes, were measured.

### Statistical analyses

All statistical analyses were performed using IBM SPSS Statistics version 20.0 (SPSS Inc., Chicago, IL, USA). Prior to hypothesis testing, data normality was assessed using the Shapiro–Wilk test. Based on the results of these normality assessments, parametric or nonparametric test procedures were applied. For each experimental condition (expanded visual field and light bar), pairwise comparisons were made between the initial fixed gaze condition and the subsequent six visual tasks (left, right, up, and down optokinetic stimulation; smooth pursuit at 0.4 Hz; and fixed gaze retest). When the data met parametric assumptions, paired sample t-tests were used. In cases of normality violations, the Wilcoxon signed-rank test was used as a nonparametric alternative. To control for Type I error from multiple comparisons, a Bonferroni correction was applied to the significance threshold, resulting in a corrected alpha level of *p* ≤ 0.008 (0.05/6 comparisons). All statistical tests were two-tailed, and results were interpreted in terms of statistical significance within this adjusted threshold.

## Results

**Fig. 1:**
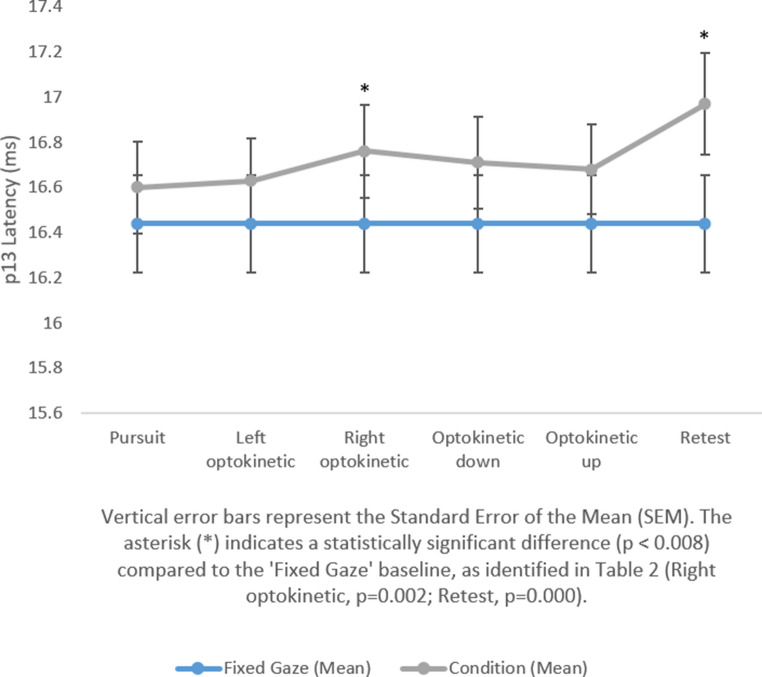
p13 latency comparison for expanded visual field

**Fig. 2:**
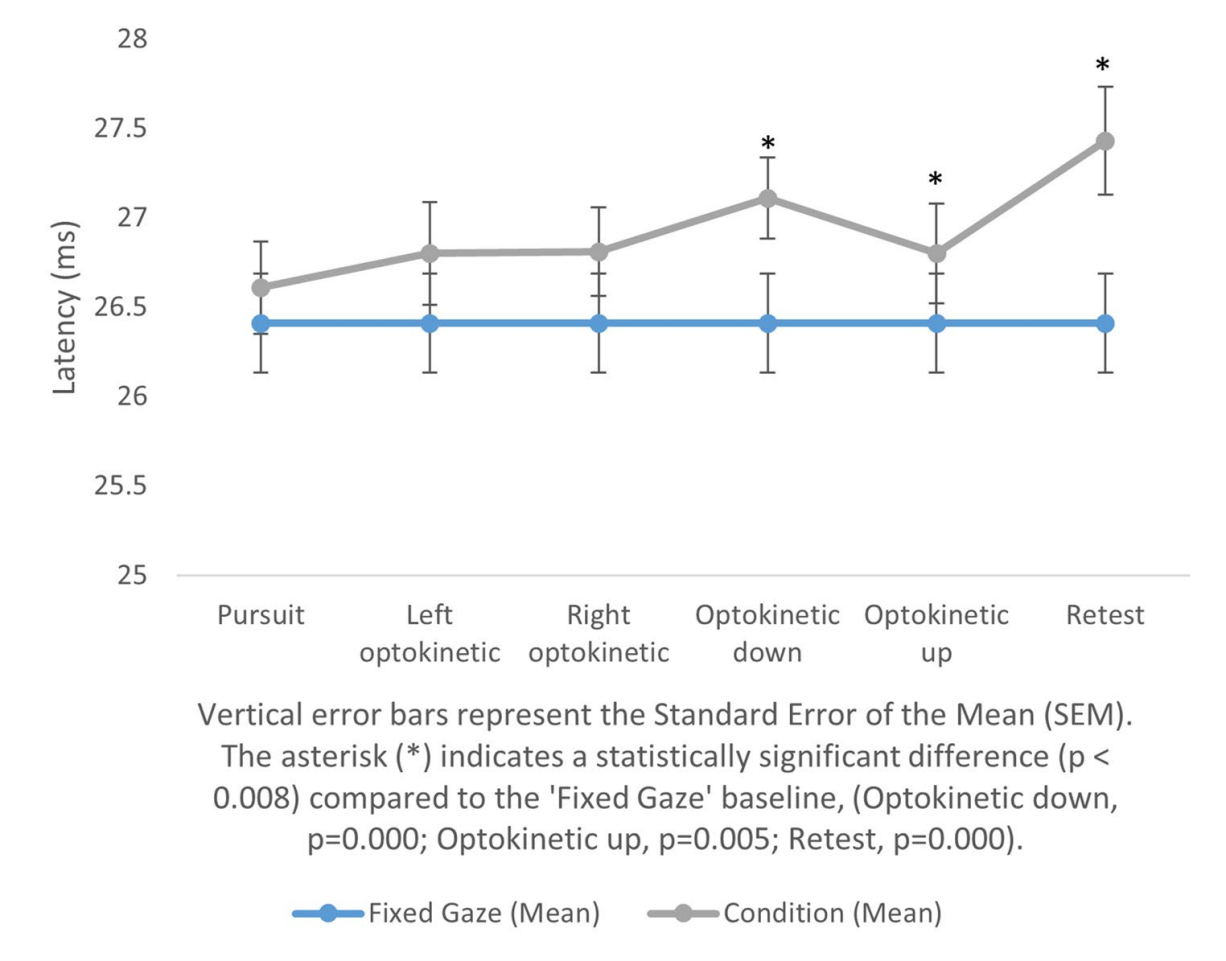
n23 latency comparison for expanded visual field

**Fig. 3:**
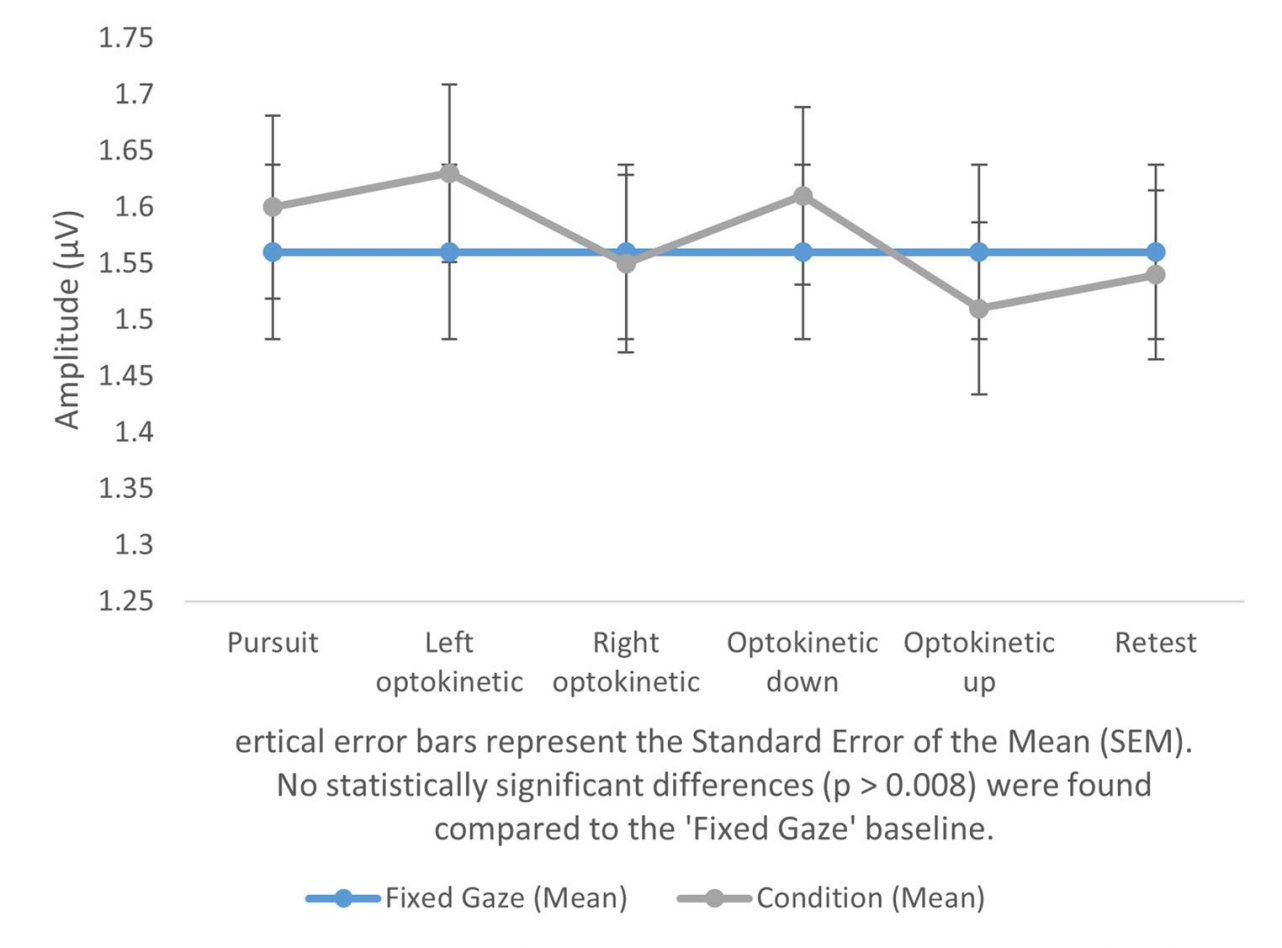
p13-n23 amplitude comparison for expanded visual field

The study found that there were no significant changes in amplitude and latency in cVEMP responses among participants when using light bars that predominantly used central vision. While no difference was observed in central vision with the light bar, significant differences emerged when peripheral vision was included using the expanded visual field. The results are presented in Table 1. These differences were particularly evident in the p13 delay in the patient’s proper optokinetic and retest comparisons with a fixed gaze, as well as in the n23 delay in the vertical changes in down and up optokinetic and retest comparisons with fixed gaze. A graphical representation of the changes is shown in Figs. 1 and 2, and Fig. 3. In tests performed using the light bar, no significant difference was found between fixed gaze and other conditions in terms of latency and amplitude (*p* < 0.008). General information on the latency and amplitude changes in cVEMP responses to central and peripheral vision, as obtained from the study, is presented in Table 2.

**Table 1:** Analysis summary of data obtained with the expanded visual field and light bar

**Expanded visual field** (***n* = 64)**	**p13 latency**	**n23 latency**	**amplitude**
Fixed gaze-pursuit	–	–	–
Fixed gaze-left optokinetic	–	–	–
Fixed gaze-right optokinetic	*	–	–
Fixed gaze-optokinetic down	–	*	–
Fixed gaze-optokinetic up	–	*	–
Fixed gaze-retest	*	*	–
**Light bar** (*n* = 26)	**p13 latency**	**n23 latency**	**amplitude**
Fixed gaze-pursuit	–	–	–
Fixed gaze-left optokinetic	–	–	–
Fixed gaze-right optokinetic	–	–	–
Fixed gaze-optokinetic down	–	–	–
Fixed gaze-optokinetic up	–	–	–
Fixed gaze-retest	–	–	–
– : no significant difference * : significant difference			

**Table 2:** Analysis of assessments in the expanded visual field

**p13 latency (ms)** ***n*** **= 64**	**Fixed gaze (mean ± SD)**	**Condition** **(mean ± SD)**	**Z**	***p***	***r***
Fixed gaze-pursuit	16.44 ± 1.72	16.60 ± 1.62	−1.36	0.172	-
Fixed gaze-left optokinetic	16.44 ± 1.72	16.63 ± 1.51	−1.87	0.061	-
Fixed gaze-right optokinetic	16.44 ± 1.72	16.76 ± 1.64	−3.16	0.002*	−0.395
Fixed gaze-optokinetic down	16.44 ± 1.72	16.71 ± 1.62	−2.58	0.010	-
Fixed gaze-optokinetic up	16.44 ± 1.72	16.68 ± 1.58	−1.64	0.100	-
Fixed gaze-retest	16.44 ± 1.72	16.97 ± 1.81	−4.20	0.000*	−0.525
**n23 latency (ms)** *n* = 64	**Fixed gaze (mean ± SD)**	**Condition** **(mean ± SD)**	**Z**	**p**	**r**
Fixed gaze-pursuit	26.41 ± 2.21	26.61 ± 2.07	−1.25	0.209	-
Fixed gaze-left optokinetic	26.41 ± 2.21	26.80 ± 2.29	−2.58	0.010	-
Fixed gaze-right optokinetic	26.41 ± 2.21	26.81 ± 1.97	−2.57	0.010	-
Fixed gaze-optokinetic down	26.41 ± 2.21	27.11 ± 1.82	−3.48	0.000*	−0.435
Fixed gaze-optokinetic up	26.41 ± 2.21	26.80 ± 2.22	−2.77	0.005*	−0.346
Fixed gaze-retest	26.41 ± 2.21	27.43 ± 2.41	−4.59	0.000*	−0.574
**p13-n23 amplitude (µV)** *n* = 64	**Fixed gaze (mean ± SD)**	**Condition** **(mean ± SD)**	**t**	**p**	**Cohen’s d**
Fixed gaze-pursuit	1.56 ± 0.62	1.60 ± 0.65	−0.87	0.384	-
Fixed gaze-left optokinetic	1.56 ± 0.62	1.63 ± 0.63	−1.83	0.072	-
Fixed gaze-right optokinetic	1.56 ± 0.62	1.55 ± 0.63	0.43	0.665	-
Fixed gaze-optokinetic down	1.56 ± 0.62	1.61 ± 0.63	−1.19	0.236	-
Fixed gaze-optokinetic up	1.56 ± 0.62	1.51 ± 0.61	1.36	0.178	-
Fixed gaze-retest	1.56 ± 0.62	1.54 ± 0.60	0.51	0.609	-

## Discussion

The balance system integrates somatosensory, visual, and vestibular inputs to generate appropriate reflex motor responses. Effective navigation within dynamic environments relies on this multisensory integration [11]. However, when conflicts arise between these inputs, the brain engages sensory reweighting mechanisms. This study aimed to investigate the influence of different types of visual stimuli on vestibular motor responses, as measured by the cVEMP test, which assesses saccular function and the vestibulo-collic reflex. Visual stimuli were delivered through the VNG-optokinetic test battery on both horizontal and vertical planes, using a wide-field TV screen and a light bar, to assess the effects on peripheral and central visual fields.

Contrary to our hypothesis, based on previous studies [7–9] suggesting latency shortening and amplitude increases with wide-field visual input, our results revealed a significant prolongation in N23 latency across most visual tasks. P13 latency was prolonged only during rightward optokinetic conditions and their retests. No consistent amplitude changes were observed under either the expanded field or light bar conditions.

Prior studies by Bansu, Öztürk, and Dündar demonstrated the vection effect—a visually induced illusion of self-motion—as a factor enhancing cVEMP amplitudes [7–9]. Öztürk reported that increased amplitudes were accompanied by latency shortening on the same side [8]. These findings suggest that vection-induced motion perception enhances vestibular responsiveness, particularly in terms of amplitude. In contrast, our findings revealed no amplitude increase and, instead, showed latency prolongation, particularly in N23. This discrepancy suggests that pure visual motion, such as optokinetic stimulation, may not engage vection mechanisms as effectively, or may operate through distinct pathways. This distinction is critical, as vection is the subjective *perception* of self-motion, which may not be automatically triggered by all forms of optokinetic stimulation, especially if the stimuli are not sufficiently immersive or ‘compelling’ to override vestibular and somatosensory cues [12]. Based on these findings, it has been demonstrated that the effects of the vection phenomenon, which is a visually induced illusion of self-motion that occurs when visual stimuli dominate an individual’s field of vision, may differ from those of pure visual stimuli. Vection-related symptoms (e.g., nausea, postural instability, headaches), collectively known as “cybersickness” [3, 8], further highlight the complexity of visually induced vestibular responses, especially in the rehabilitation of pure otolith disorders [13]. Bonsu et al. also define the amount of vection effects as having an impact on vestibular responses [7]. These findings also demonstrate that visual stimuli induce an increase in amplitude of reactions, resulting in the formation of the vection effect, without affecting latencies. However, in some cases, such as optokinetic stimuli, visual stimuli cause increases in latencies.

Central visual field stimulation using a light bar did not yield significant changes in cVEMP parameters. This aligns with prior findings indicating that peripheral visual input plays a more dominant role in modulating vestibular responses [14]. These results support the idea that peripheral motion more strongly influences vestibulo-collic reflexes and drives sensory reweighting mechanisms more effectively than central vision [15, 16]. These also strongly support the foundational concept that the peripheral visual field holds dominance over the central field in driving the perception of self-motion and associated postural adjustments [17].

The observed latency prolongation in conditions with expanded-field, peripheral visual input further reinforces the dominant role of peripheral vision in vestibular modulation. Unlike previous studies that used irregular motion stimuli, our optokinetic stimuli consisted of highly regular and repetitive patterns. This may have led to rapid visual adaptation, possibly through preprogrammed saccadic responses—mechanisms known to influence outcomes in vestibular rehabilitation. This high degree of predictability in the stimulus may have induced rapid sensory adaptation, a mechanism consistent with predictive coding frameworks where highly predictable inputs are processed differently or ‘explained away’ compared to novel sensory ‘surprises’ [18].

Inter-individual variability, including differences in SCM muscle tone, attentional state, or neural conduction velocity, may have influenced the observed latency values. Previous evidence shows that cVEMP amplitude is sensitive to SCM activity levels [19]. Moreover, the attentional demand imposed by optokinetic stimulation tasks (e.g., tracking a moving point) may have introduced cognitive modulation of reflex responses [20, 21]. These findings align with literature suggesting that multisensory integration under sensory conflict is susceptible to attentional network modulation, involving prefrontal and parietal areas. Indeed, top-down cognitive processes, particularly those related to spatial attention and task focus, are known to modulate the gain of brainstem-level vestibular reflexes, providing a plausible pathway for the cognitive modulation of cVEMP responses [22].

Crucially, the discrepancy between P13 and N23 responses raises questions about the respective neural generators and their susceptibility to cognitive modulation. Current evidence suggests that P13 reflects the early activation of the saccule-inferior vestibular nerve–brainstem (lateral vestibular nucleus)–vestibulospinal tract–SCM motor neuron pathway [23]. In contrast, N23 may represent the end of the inhibition of the SCM as well as the efferent reflection of afferent signals processed in the vestibular nuclei and cerebellar or cortical modulation [23]. The observed N23 latency prolongation without a corresponding N13 shift can support a potential dissociation in the functional origins or modulation sensitivity of these waveforms. This dissociation aligns with the established model of P13 as a rapid, oligosynaptic brainstem reflex and N23 as a more complex, polysynaptic response that is more susceptible to modulation from higher-order centres, including cortical and cerebellar inputs [24]. This may reflect the differential susceptibility of these generators to visual-cognitive interference.

This study contributes to the growing body of research on visual-vestibular integration by showing that repetitive visual motion stimuli can modulate cVEMP latencies—particularly prolonging N23—despite the absence of confirmed vection experiences. The dissociation between latency changes and amplitude stability suggests the presence of complex modulatory mechanisms, possibly involving timing or inhibitory pathways. Future research should incorporate vection-specific measures, cognitive load manipulations, and neuroimaging to clarify the perceptual and neural underpinnings of these effects and to inform vestibular rehabilitation strategies targeting otolith-related dysfunction.

### Limitations of the study

A primary limitation of this study is the absence of a subjective measure for vection. While we discuss our findings in the context of prior vection-related literature, we cannot definitively conclude whether our stimuli induced a vection illusion. Therefore, our interpretations linking the observed N23 latency delays to a *lack* of vection are speculative. Future studies should incorporate subjective ratings (e.g., a Vection Rating Scale) alongside cVEMP measures to clarify this relationship.
